# Insulin Resistance and Cancer

**DOI:** 10.1111/1753-0407.70064

**Published:** 2025-02-26

**Authors:** Zachary Bloomgarden

**Affiliations:** ^1^ Department of Medicine, Division of Endocrinology, Diabetes and Bone Disease Icahn School of Medicine New York New York USA

It has long been clear that Type 2 diabetes is associated with an increased likelihood of the development of a variety of malignancies. The Shanghai Standardized Diabetes Management System data set of more than 400 000 persons from 2011 to 2018 showed a 10% increase in overall malignancy rates over those in the nondiabetic population, with a particularly high risk of cancers of the pancreas [1], thyroid [2], bladder, kidney, breast, colorectum, and liver compared with the general population, and with a greater relative increase in younger persons [3]. Several mechanisms have been proposed for this association [4]. Hyperglycemia itself might predispose individuals to cancer [5, 6]. Obesity underlies the majority of instances of Type 2 diabetes and is associated with malignancy, with adipose tissue secretion of inflammatory cytokines and leptin, decreased production of adiponectin, and, particularly in postmenopausal women, adipose tissue estrogen production playing roles in specific tissues. An attractive hypothesis of the underlying mechanism for all of these is that insulin resistance itself gives rise to malignancy [7].

Certainly, it is plausible that insulin resistance may be a major underlying cause of cancer development. Insulin has mitogenic effects, which may particularly manifest in insulin resistance with hyperinsulinemia, and insulin may be pro‐angiogenic, leading to an anti‐apoptotic effect in DNA‐damaged cells, furthering carcinogenesis; features of these mechanisms have been shown in breast cancer models, with the insulin receptor substrate receptor (IRS)‐1 showing high expression, and with adipose tissue secretory proteins such as leptin stimulated by insulin and having mitogenic, angiogenic, and anti‐apoptotic effects, while adiponectin levels are suppressed by insulin and lowered in obesity, leading to opposing effects [8]. In breast cancer development, factors related to insulin resistance produced in systemic adipose tissue may have endocrine effects, adipocytes in the tumor capsule would have paracrine effects, and factors directly produced by tumor cells could have autocrine effects in cancer promotion [9]. In the development of pancreatic cancer, insulin might act on pancreatic acinar cells to increase digestive enzyme production, leading to inflammation in pancreatic exocrine tissue, while insulin might also directly increase pancreatic cell mitosis and potentiate metaplasia [9].

Proxy measures of insulin resistance have been used to support this concept. In a study based on nearly 400 000 persons in the UK Biobank, the triglyceride‐glucose‐BMI product, an insulin resistance measure based on the metabolic syndrome, and the ratio of triglyceride to HDL cholesterol were analyzed to determine the relationship between insulin resistance and esophageal cancer risk [10], showing adenocarcinoma to track with features of insulin resistance, although esophageal squamous cell carcinoma risk was greatest in individuals with lower levels of insulin resistance. A 9‐year follow‐up from the UK Biobank failed to show an association of lung cancer development with the triglyceride‐glucose index [11], further showing the heterogeneity of these relationships among different forms of cancer. The triglyceride‐glucose‐BMI index and the metabolic syndrome index are in part based on glucose measures, and as such might reflect the association of cancer risk with diabetes, but the triglyceride/HDL ratio is a well‐recognized marker of insulin resistance not including measures of glycemia [12], suggesting a direct role of insulin resistance, with potential mechanisms including the proinflammatory and pro‐proliferative effects of hyperinsulinemia, insulin signaling via the human epidermal growth factor receptor 2 (HER2), and effects of insulin resistance (as well as obesity) on increasing gastroesophageal reflux [11]. In a population with high hepatitis B prevalence in Northern China, a 22‐year follow‐up showed hyperglycemia and, even more strongly, hyperinsulinemia and elevation in the HOMA‐IR measure of insulin resistance to be associated with a 2‐3‐fold increase in the development of hepatocellular carcinoma [13]. A meta‐analysis of 31 publications involving more than 6.5 million people with over 62 000 cases of lung cancer showed significant correlation with HOMA‐IR, a less strong correlation with diabetes, but negative correlation with BMI and no correlation with obesity or with a metabolic syndrome index [14]. In a 10‐year follow‐up of colorectal cancer incidence among more than 300 000 persons, an association was shown with triglyceride/HDL, appearing to show a protective effect of low triglyceride/HDL rather than a linear relationship across the spectrum of insulin sensitivity [15].

This latter finding suggests that measures to improve insulin sensitivity may ultimately reduce malignancy across populations. Reduction in cancer development has not been shown with diabetes medications [5]. However, a study of 346 627 persons with > 10‐year follow‐up showed protective effects of self‐reported physical activity [16], and a 6‐year UK Biobank follow‐up of 86 556 persons who wore an accelerometer for 1 week showed a linear reduction in the malignancy hazard rate with increasing overall physical activity and with increasing step count [17] (Figure 1).

**FIGURE 1:**
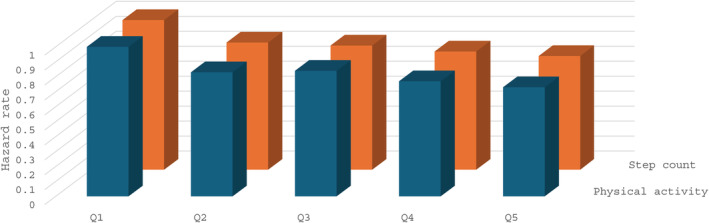
Malignancy hazard rate versus accelerometer‐measured activity in UK Biobank participants. Redrawn from data in [17].

Thus, diabetes and obesity are associated with many forms of malignancy, with insulin resistance potentially playing a role. Cancer incidence is predicted to double over the next five decades [18]. Measures on a population basis to improve insulin sensitivity may ultimately reduce this growing burden of cancer.

## Conflicts of Interest

The author declares no conflicts of interest.

## References

[1] B. Shen , Y. Li , C. S. Sheng , et al., “Association Between Age at Diabetes Onset or Diabetes Duration and Subsequent Risk of Pancreatic Cancer: Results From a Longitudinal Cohort and Mendelian Randomization Study,” Lancet Regional Health ‐ Western Pacific 30 (September 2022): 100596. 36419740 10.1016/j.lanwpc.2022.100596 PMC9677075

[2] T. Hou , Y. Li , Q. Yan , et al., “The Interaction Effect Between BMI, Diabetes and Age at Diabetes Onset on the Risk of Thyroid Cancer: A Population‐Based Cohort Study in Shanghai, China,” Diabetes, Obesity & Metabolism 26, no. 9 (2024): 3988–3997. 10.1111/dom.15746 38978180

[3] Y. Li , J. Tian , T. Hou , et al., “Association Between Age at Diabetes Diagnosis and Subsequent Incidence of Cancer: A Longitudinal Population‐Based Cohort,” Diabetes Care 47, no. 3 (March 2024): 353–361. 38237119 10.2337/dc23-0386 PMC10909688

[4] Y. Handelsman , D. Leroith , Z. T. Bloomgarden , et al., “Diabetes and Cancer—An AACE/ACE Consensus Statement,” Endocrine Practice 19, no. 4 (2013): 675–693. 23978590 10.4158/EP13248.CS

[5] Y. Bi , J. Lu , W. Wang , et al., “Cohort Profile: Risk Evaluation of Cancers in Chinese Diabetic Individuals: A Longitudinal (REACTION) Study,” Journal of Diabetes 6, no. 2 (2014): 147–157. 24237858 10.1111/1753-0407.12108

[6] N. Parekh , Y. Lin , R. B. Hayes , J. B. Albu , and G. L. Lu‐Yao , “Longitudinal Associations of Blood Markers of Insulin and Glucose Metabolism and Cancer Mortality in the Third National Health and Nutrition Examination Survey,” Cancer Causes & Control 21, no. 4 (2010): 631–642. 20094767 10.1007/s10552-009-9492-y PMC3817266

[7] T. Wang , G. Ning , and Z. Bloomgarden , “Diabetes and Cancer Relationships,” Journal of Diabetes 5, no. 4 (2013): 378–390. 23574745 10.1111/1753-0407.12057

[8] D. R. Rose and L. Vona‐Davis , “The Cellular and Molecular Mechanisms by Which Insulin Influences Breast Cancer Risk and Progression,” Endocrine‐Related Cancer 19, no. 6 (2012): R225–R241, 10.1530/erc-12-0203. 22936542

[9] A. M. Y. Zhang , Y. H. Xia , J. S. H. Lin , et al., “Hyperinsulinemia Acts via Acinar Insulin Receptors to Initiate Pancreatic Cancer by Increasing Digestive Enzyme Production and Inflammation,” Cell Metabolism 35, no. 12 (December 2023): 2119–2135.e5. 37913768 10.1016/j.cmet.2023.10.003

[10] C. Yang , W. Cheng , P. S. Plum , J. Köppe , I. Gockel , and R. Thieme , “Association Between Four Insulin Resistance Surrogates and the Risk of Esophageal Cancer: A Prospective Cohort Study Using the UK Biobank,” Journal of Cancer Research and Clinical Oncology 150, no. 8 (August 2024): 399, 10.1007/s00432-024-05919-8. 39180548 PMC11344731

[11] L. Wang , S. Si , J. Li , et al., “Triglyceride‐Glucose Index Is Not Associated With Lung Cancer Risk: A Prospective Cohort Study in the UK Biobank,” Frontiers in Oncology 11 (November 2021): 774937, 10.3389/fonc.2021.774937. 34869022 PMC8635521

[12] M. R. Salazar , H. A. Carbajal , W. G. Espeche , et al., “Comparison of the Abilities of the Plasma Triglyceride/High‐Density Lipoprotein Cholesterol Ratio and the Metabolic Syndrome to Identify Insulin Resistance,” Diabetes & Vascular Disease Research 10, no. 4 (2013): 346–352. 23624761 10.1177/1479164113479809 PMC5858929

[13] J. Yin , N. D. Freedman , Y. Liu , et al., “Associations Between Serum Glucose, Insulin, Insulin Resistance and the Risk of Incident Primary Liver Cancer or Chronic Liver Disease Mortality: A Nested Case‐Control Study,” British Journal of Cancer 128, no. 2 (2023): 275–284. 36496451 10.1038/s41416-022-02042-8 PMC9902537

[14] J. Liu , R. Wang , S. Tan , X. Zhao , and A. Hou , “Association Between Insulin Resistance, Metabolic Syndrome and Its Components and Lung Cancer: A Systematic Review and Meta‐Analysis,” Diabetology and Metabolic Syndrome 16, no. 1 (March 2024): 63. 38468310 10.1186/s13098-024-01308-w PMC10926619

[15] M. Son , S. Y. Moon , M. Koh , Y. Kang , and J. Y. Lee , “Association Between Surrogate Markers of Insulin Resistance and the Incidence of Colorectal Cancer in Korea: A Nationwide Population‐Based Study,” Journal of Clinical Medicine 13, no. 6 (2024): 1628, 10.3390/jcm13061628. 38541854 PMC10971512

[16] D. Ding , J. Van Buskirk , B. Nguyen , et al., “Physical Activity, Diet Quality and All‐Cause Cardiovascular Disease and Cancer Mortality: A Prospective Study of 346 627 UK Biobank Participants,” British Journal of Sports Medicine 56, no. 20 (2022): 1148–1156, 10.1136/bjsports-2021-105195. 35811091

[17] A. H. Shreves , S. R. Small , R. Walmsley , et al., “Amount and Intensity of Physical Activity and Risk of Incident Cancer in the UK Biobank,” *medRxiv*. Published ahead of print, December 4, 2023.

[18] I. Soerjomataram and F. Bray , “Planning for Tomorrow: Global Cancer Incidence and the Role of Prevention 2020‐2070,” Nature Reviews. Clinical Oncology 18, no. 10 (2021): 663–672. 10.1038/s41571-021-00514-z 34079102

